# Integrative analysis of Iso-Seq and RNA-seq reveals dynamic changes of alternative promoter, alternative splicing and alternative polyadenylation during Angiotensin II-induced senescence in rat primary aortic endothelial cells

**DOI:** 10.3389/fgene.2023.1064624

**Published:** 2023-01-19

**Authors:** Haimei Wen, Wei Chen, Yu Chen, Gang Wei, Ting Ni

**Affiliations:** ^1^ Collaborative Innovation Center of Genetics and Development, School of Life Sciences, Human Phenome Institute, Fudan University, Shanghai, China; ^2^ Ministry of Education Key Laboratory of Contemporary Anthropology, School of Life Sciences, Fudan University, Shanghai, China

**Keywords:** rat primary aortic endothelial cells senescence, Iso-Seq, RNA-seq, Angiotensin II, alternative promoter, alternative splicing, alternative polyadenylation

## Abstract

In eukaryotes, alternative promoter (AP), alternative splicing (AS), and alternative polyadenylation (APA) are three crucial regulatory mechanisms that modulate message RNA (mRNA) diversity. Although AP, AS and APA are involved in diverse biological processess, whether they have dynamic changes in Angiotensin II (Ang II) induced senescence in rat primary aortic endothelial cells (RAECs), an important cellular model for studying cardiovascular disease, remains unclear. Here we integrated both PacBio single-molecule long-read isoform sequencing (Iso-Seq) and Illumina short-read RNA sequencing (RNA-seq) to analyze the changes of AP, AS and APA in Ang II-induced senescent RAECs. Iso-Seq generated 36,278 isoforms from 10,145 gene loci and 65.81% of these isoforms are novel, which were further cross-validated by public data obtained by other techonologies such as CAGE, PolyA-Seq and 3′READS. APA contributed most to novel isoforms, followed by AS and AP. Further investigation showed that AP, AS and APA could all contribute to the regulation of isoform, but AS has more dynamic changes compared to AP and APA upon Ang II stimulation. Genes undergoing AP, AS and APA in Ang II-treated cells are enriched in various pathways related to aging or senescence, suggesting that these molecular changes are involved in functional alterations during Ang II-induced senescence. Together, the present study largely improved the annotation of rat genome and revealed gene expression changes at isoform level, extending the understanding of the complexity of gene regulation in Ang II-treated RAECs, and also provided novel clues for discovering the regulatory mechanism undelying Ang II caused vascular senescence and diseases.

## Introduction

Rat has become an important model in the field of cardiovascular diseases (CVDs) such as hypertension, cardiac hypertrophy, and heart failure (Doggrell and Brown, 1998). However, the Rat genome is not well annotated compared to that of human and mouse (Wang et al., 2019). Endothelial cell (EC) senescence appears to be the first step in a series of events that culminate with the development of cardiovascular pathologies (Carracedo et al., 2018). The renin-angiotensin system (RAS) plays an important role in vascular biology and Angiotensin II (Ang II) is a principal effector of RAS. Ang II serves as an important signaling molecule involved in atherogenic stimuli and is known to promote cellular senescence and aging (Shan et al., 2008). Ang II can stimulate cell migration (Nadal et al., 2002) and induce angiogenesis *via* upregulation of vascular endothelial growth factor (VEGF) (Suganuma et al., 2005). Ang II is also involved in the pathogenesis of endothelial dysfunction (ED) through multiple signaling pathways, including angiotensin type 1 receptor (AT1R)-mediated NADPH oxidase (Nox)/reactive oxygen species (ROS) (Nguyen Dinh Cat et al., 2012). Most CVDs, as well as age-related cardiovascular changes, are associated with increases in oxidative stress, due to increased generation and/or decreased metabolism of ROS (Ding et al., 2020). Ang II can also activate NF-κB pathway, which in turn induced the expression of pro-inflammatory genes (Kim et al., 2012) and inflammation-related genes [e.g., VCAM-1, iNOS, COX-2 and IL-6] (Yang et al., 2018). These inflammatory factors ususlly correlated with series of senescence-associated secretory phenotypes (SASP). Previous studies have investigated the events of Ang II-induced senescence of ECs from molecular perspective (Forrester et al., 2018), however, no systematic analysis of the dynamic changes at the isoform levels has been performed.

Transcription is a highly regulated process, and stress-induced changes in gene transcription have been shown to play a major role in stress response and adaptation (Vilborg et al., 2017). Alternative promoter (AP), alternative splicing (AS), and alternative polyadenylation (APA) are three key steps that determine the final products of gene transcription, which greatly increase the transcriptome complexity (Anvar et al., 2018; Demircioğlu et al., 2019). Alternative promoter generates transcription start sites (TSSs) from different genomic locations and results in gene isoforms with distinct 5′untranslated regions (5′UTRs) (Ayoubi and Van De Yen, 1996). Alternative promoters are particularly fascinating due to their abnormal usage in several diseases (Singer et al., 2008). Previous analysis shows that the p21-coding gene has two transcripts, p21B and p21C, derived from alternative promoters and exhibit different functions. While p21C induces cell cycle arrest, p21B appears to stimulate apoptosis (Landry et al., 2003).

In eukaryotic genomes, AS is a prevalent phenomenon that can expand the protein pool without increasing the number of genes (Roy et al., 2013). This helps to promote the evolution of complex functional transcriptomes that can control a range of cellular, molecular, and developmental events. AS is emerging as a critical contributor to senescence and aging (Kwon et al., 2021). For example, PRPF19 downregulation changes the MDM4 splicing isoform from MDM4-FL to MDM4-S, which induces p53-p21-dependent cellular senescence (Yano et al., 2021).

APA is a phenomenon that RNA molecules with different 3′ends originate from distinct polyadenylation sites (PASs) of a single gene, which can influence the translation efficiency, stability, and localization of an mRNA (Tian and Manley, 2016; Chen et al., 2017). Multiple studies had showed that APA-mediated 3′UTR length changes were dynamically regulated during cellular senescence and could serve as a novel player in regulating senescence (Chen et al., 2018; Shen et al., 2019; Schwich et al., 2021). Although AP, AS or APA have been separately reported to function in replicative senescence or age-related diseases, whether they are involved in Ang II-induced RAECs senescence, an important and widely used cellular model in cardiovascular disease, remains unkown.

Traditional short-read RNA-seq is commonly used for quantifying genome-wide gene expression and AS events due to its high throughput (Wang et al., 2009). But there is still a challenge for short reads [typically 100 to 250 base pairs (bp)] to infer the structure of full-length transcripts, which are often several thousand bases long (Williams et al., 2014). Single-molecule real-time (SMRT) Iso-Seq using the Pacific Biosciences (PacBio) platforms can capture full-length transcripts, making it possible to refine genome annotation and discover novel transcripts and fusion genes. However, the base sequencing error rate for Iso-Seq is relatively higher than RNA-seq, so high-quality RNA-seq reads are often used to correct errors in Iso-Seq such as FMLRC (Wang J. R. et al., 2018), proovread (Förster et al., 2014), LoRDEC (Rivals and Salmela, 2014). The quantification accuracy for Iso-Seq is also lower than RNA-seq (Wang et al., 2019). Therefore, a combination of both Iso-Seq and RNA-seq would improve the performance at both qualitative and quantitative levels.

In this study, we systematically characterized the transcriptome diversity and changes in RAECs before and after Ang II treatment. We first used the PacBio Iso-Seq approach (Gordon et al., 2015) to generate full-length isoforms. We found widespread transcript diversity, with the detection of novel transcripts not in existing rat genomic annotations, and used short-read RNA-seq to cross-validate and complement the Iso-Seq result. We discovered many novel and differential alternative AP, AS and APA events between Control and Ang II-induced cells. Our results widen the research of Ang II-induced senescence in RAECs and also contribute to the investigation of transcriptome complexity in the research field of CVDs.

## Materials and methods

### Culture of rat primary aorta endothelial cells (RAECs)

Rat primary aortic endothelial cells (RAECs) were isolated from male rats and those with the purity greater than 95% (determined by both positive staining for CD31 and morphologically based on their classical “hill and valley” appearance) were kept for the subsequent study, as performed according to the methods described previously (Yang et al., 2018). Cells that experienced three to five passages were used for Ang II stimulation experiments. Quantified RAECs were treated with Ang II (2 μM) or vehicle (sodium chloride) for 48 hours (h) as described before (Yang et al., 2018).

### RNA-seq library construction

PolyA^+^ RNA was enriched by oligo (dT)25 Dynabeads (Invitrogen) from total RNA. The dUTP-based strand-specific RNA-seq libraries for control and Ang II-induced RAECs were constructed according to the method previously described (Yang et al., 2020). RNA-seq libraries were sequenced on Illumina HiSeq platform, and paired-end reads of 150 nucleotide (nt) lengths were obtained.

### PacBio sequencing library construction

PolyA^+^ RNA after double-stranded cDNA preparation, PCR amplification, product purification with AMPurePB magnetic beads, verification of DNA size, followed by qualitative and quantitative analysis using a Bioanalyzer. The resulting double-stranded DNA was used to generate the SMRTbell™ libraries using PacBio Template Prep Kit. SMRTbell templates are then sequenced in the PacBio Sequel System.

### Iso-seq data processing

We performed initial data processing using IsoSeq v3 pipeline (IsoSeq/isoseq-clustering.md at master·PacificBiosciences/IsoSeq·GitHub) for raw subreads. The circular consensus sequencing (CCS) reads were obtained through the consensus generation step. Then, we determine whether they are classified as full-length reads based on the presence or absence of the 5′and 3′primers, and use lima v2.0.0 (https://lima.how/) to remove the primers. The obtained full-length reads, were refined by trimming poly (A) tails and removing concatemer, then full-length non-concatemer (FLNC) reads are finally obtained. To find transcript clusters, isoform-level clustering algorithm ICE (Iterative Clustering for Error Correction) (Gordon et al., 2015) was applied to all full-length (FL) transcripts to obtain the consensus sequence for each cluster. Quiver (Chin et al., 2013) was used for error correction to obtain high-quality (HQ) (accuracy, ≥99%) isoforms. To get non-redundant isoforms, HQ isoforms were mapped to rat genome (mRatBN7.2) using minimap2 (Li, 2018) with parameters -ax splice -uf--secondary = no -C5 -O6,24 -B4, and collapsed by a third-party module Cupcakes Python script (collapse_isoforms_by_sam.py) with the following parameters “-c 0.99 -i 0.85”. The 5′degraded isoforms were filtered away because the Clontech SMARTer cDNA kit used to create the full-length cDNA does not do cap trap, these may be 5′RNA degradation products and not biologically meaningful. In that case, we filter them away according to the method described in Cupcake (https://github.com/Magdoll/cDNA_Cupcake/wiki/Cupcake:-supporting-scripts-for-Iso-Seq-after-clustering-step#filtersubset). Then the resulted isoforms were finally annotated using SQANTI3 (Tardaguila et al., 2018). SQANTI3 marked the internal priming resulted PAS by identifying downstream six consecutive “A” or the percentage of “A” is greater than 60% in the 20 nucleotides downstream of the potential PAS, the transcript is considered to be an IntraPriming transcript and is filtered out.

The coding potential of the resulted isoforms was also predicted with SQANTI3 which uses GeneMarkS-T (GMST) algorithm (Tang et al., 2015). GeneMarkS-T utilizes iterative self-training and a hidden semi-Markov model to predict coding regions in eukaryotic transcripts.

All FLNC reads were merged and the FMLRC (Wang J. R. et al., 2018) was then used for error correction with RNA-seq to get high-quality FLNC reads. The corrected FLNC reads were then mapped to mRatBN7.2 genome using minimap2 (Li, 2018) with parameters -ax splice -uf--secondary = no -C5 -O6,24 -B4, then using the sam_to_gff3.py from cDNA_Cupcake to generate an FLNC gff3 file. This file was then compared with non-redundant HQ isoforms using inhouse scripts to calculate the FLNC reads supporting HQ isoforms in Control and Ang II samples independently.

### Transcript quantification and differential expression analysis

First, RNA-seq raw reads were cleaned by trimming using Cutadapt (Martin, 2011) with the default parameter. The clean reads were mapped to the mRatBN7.2 reference genome using STAR (version 2.7.7a) (Dobin et al., 2013) with parameters--twopassMode Basic--outFilterMultimapNmax 1 --outSAMstrandField intronMotif. Differential expression analysis between two conditions was performed using the DESeq2 R package (version 1.30.1) (Love et al., 2014). The resulting *p* values were adjusted using Benjamin and Hochberg’s approach for controlling for the false discovery rate (FDR). Genes with an adjusted *p*-value <0.05, and |FoldChange (FC)| > 1.5 identified by DESeq2 (Love et al., 2014) were assigned as differentially expressed. Transcripts per million (TPM) of reference transcriptome defined using Iso-Seq was used in calculating the expression level of transcripts by Salmon (v1.8.0) (Patro et al., 2017).

### Functional annotation and enrichment analysis

For differentially expressed genes (DEGs) and differentially spliced genes, GO and KEGG enrichment analysis were performed with clusterProfiler (Yu et al., 2012), which supports statistical analysis and visualization of functional profiles for genes and gene clusters.

### Alternative promoter analysis

Active promoter sites were identified by *proActiv* (v0.1.0) (Demircioğlu et al., 2019) based on transcriptome generated by Iso-Seq. Short-reads of RNA-seq were aligned to the reference genome using STAR (Dobin et al., 2013). The aligned RNA-seq bam files and GTF files generated from Iso-Seq were used as input by *proActive* to estimate promoter activities in each sample. We divided the promoter set into three categories depending on their relative promoter activity calculated by *proActive*, major, minor and inactive promoters. Promoters with the highest relative activity for each gene across the sample cohort were defined as major promoters. Besides, promoters with relative promoter activity less than 0.1 constitute inactive promoters whereas the rest promoters of a gene constitute minor promoters. Known promoter was defined if known genome annotation also uses this promoter. Novel promoter was defined only if Iso-Seq isoforms use this promoter. Differentially regulated promoters (DRPs) were identified using DESeq2. The resultant *p* values were adjusted using BH approach for controlling the false discovery rate (FDR). The promoters with an activity change level of |FoldChange| > 1.5 and adjusted *p*-value <0.05 were considered significant DRPs. We identified alternative promoters (APs) by considering both promoter expression and promoter activity. The criteria were as follows: (1) mean relative promoter activity >0.1 and were significantly changed between two conditions (FDR <0.05, BH adjusted); (2) |Fold Change| > 1.5 of promoter expression. (3) Gene expression FPKM >1.

### Alternative splicing and specific isoform analysis

Through the use of SUPPA2 (Trincado et al., 2018), a tool designed to find splicing events and determine whether there are differences between samples, alternative splicing patterns were found with the PacBio full-length transcripts. By scanning the ‘exon’ lines and categorizing each local event as a particular splicing type, the SUPPA2 “generateEvents” command was used to create local alternative splicing events from our high-quality PacBio transcript annotation. Then, using the Salmon (Patro et al., 2017) quantification results, the SUPPA2 “psiPerEvent” command was used to quantify the percentage of spliced-in (PSI) values for each local alternative splicing event. Differential expression of AS events was screened out with the adjusted *p*-value <0.05.

### Alternative polyadenylation analysis

PAS cluster was using with modified TAPIS 1.2.1 (Abdel-Ghany et al., 2016) (https://bitbucket.org/comp_bio/tapis/src/master/) scripts that PASs within 50 base pair (bp) were clustered as one PAS. APA analysis between two conditions was performed by using the algorithm of DaPars (Xia et al., 2014). As DaPars predicts the proximal PAS using RNA-seq and Iso-Seq could provide validated PAS, we therefore supplied Iso-Seq supported PAS for APA analysis. These PAS were used as prior knowledge using a modified DaPars algorithm to calculate the distal PAS usage index (PDUI) based on RNA-seq coverage, and then Fisher exact test was used to determine differential APA events between the two groups, and the criteria for defining differential APA events between two groups were consistent with DaPars.

### 3′READS and Poly (A)-seq data analysis

3′READS and Poly (A)-seq reads were mapped to mRatBN7.2 genome using Bowtie 2 with the default parameters (Langmead and Salzberg, 2012). Reads start pos were considered as potential PAS. PAS with at least five reads support is considered a potential validated PAS.

### CAGE data analysis

CAGE reads were mapped to mRatBN7.2 genome using Bowtie 2 (Langmead and Salzberg, 2012) with default parameters. Then peak calling was performed using MACS2 (Zhang et al., 2008). The resultant files with suffix of.treat_pileup.bdg and.summits.bed were used for further analysis.

## Results

### Overall statistics of the Iso-Seq profiling on RAECs

RNA-seq is widely used for the quantification of gene expression and AS events. However, it is still challenging to accurately identify full-length splicing isoforms using RNA-seq alone (Abdel-Ghany et al., 2016). To reveal the complexity of the transcriptome of rat primary aortic endothelial cells (RAECs) and discover dynamic changes of alternative promoter (AP), alternative splicing (AS) and alternative polyadenylation (APA) in Ang II-induced endothelial cell senescence, we sequenced the transcriptome of RAECs before (Control) and after Ang II treatment using both PacBio Iso-Seq and Illumina RNA-seq techniques.

We used PacBio’s official pipeline, IsoSeq3, to filter and process the raw sequencing files (details see: https://github.com/PacificBiosciences/IsoSeq/blob/master/isoseq-clustering.md). As a result, a total of 27,668,459 raw subreads with an average length of 1,838 bp were obtained in Control sample and 33,151,284 raw subreads with an average length of 1,700 bp in Ang II treatment samples (Table 1). The PacBio official pipeline we adopted for the analysis of raw data includes circular consensus sequencing (CCS) reads calling, primer removal and demultiplexing, and the refining steps (Supplementary Figure S1) (Details in Methods section). After processing with these steps, we got 1,469,285 FLNC reads with an average length of 2,361 bp in the Control sample and 1,676,143 FLNC with an average length of 2,486 bp in the Ang II sample, respectively (Table 1). Then, polish step was performed on all the full-length transcripts to generate consensus isoforms. It created a consensus isoform for each cluster after classifying them into groups based on their sequence similarity. Subsequently, the full insert sequence that had not been tested was aligned to the adjusted to produce high-quality (HQ) isoforms (125,900) with a mean length of 2,603 from the consensus isoform and low quality (LQ) isoforms (5,323) with a mean length of 2,807 (Table 1). To get non-redundant transcripts, HQ isoforms were mapped to rat genome (mRatBN7.2) using minimap2 (Li, 2018) and collapsed with a third-party module Cupcakes Python script (collapse_isoforms_by_sam.py). Then we got 36,278 transcripts derived from 10,145 gene loci. These transcripts were annotated by SQANTI3 (Tardaguila et al., 2018) for further analysis.

**TABLE 1:** Summary of PacBio raw data analysis of Control and Ang II in RAECs.

Subject	Category	Control	Ang II
Subreads	Subreads number	27,668,459	33,151,284
	Average length (bp)	1838	1700
Number of CCS	Full-length non-concatemer (FLNC)	1,469,285	1,676,143
	Average length (bp)	2,361	2,486
HQ isoform	Number of polished high-quality isoforms	125,900	
	Average length (bp)	2,603	
LQ isoform	Number of polished low-quality isoforms	5,323	
	Average length (bp)	2,807	

### Full-length isoforms greatly improve rat genome annotation

**FIGURE 1:**
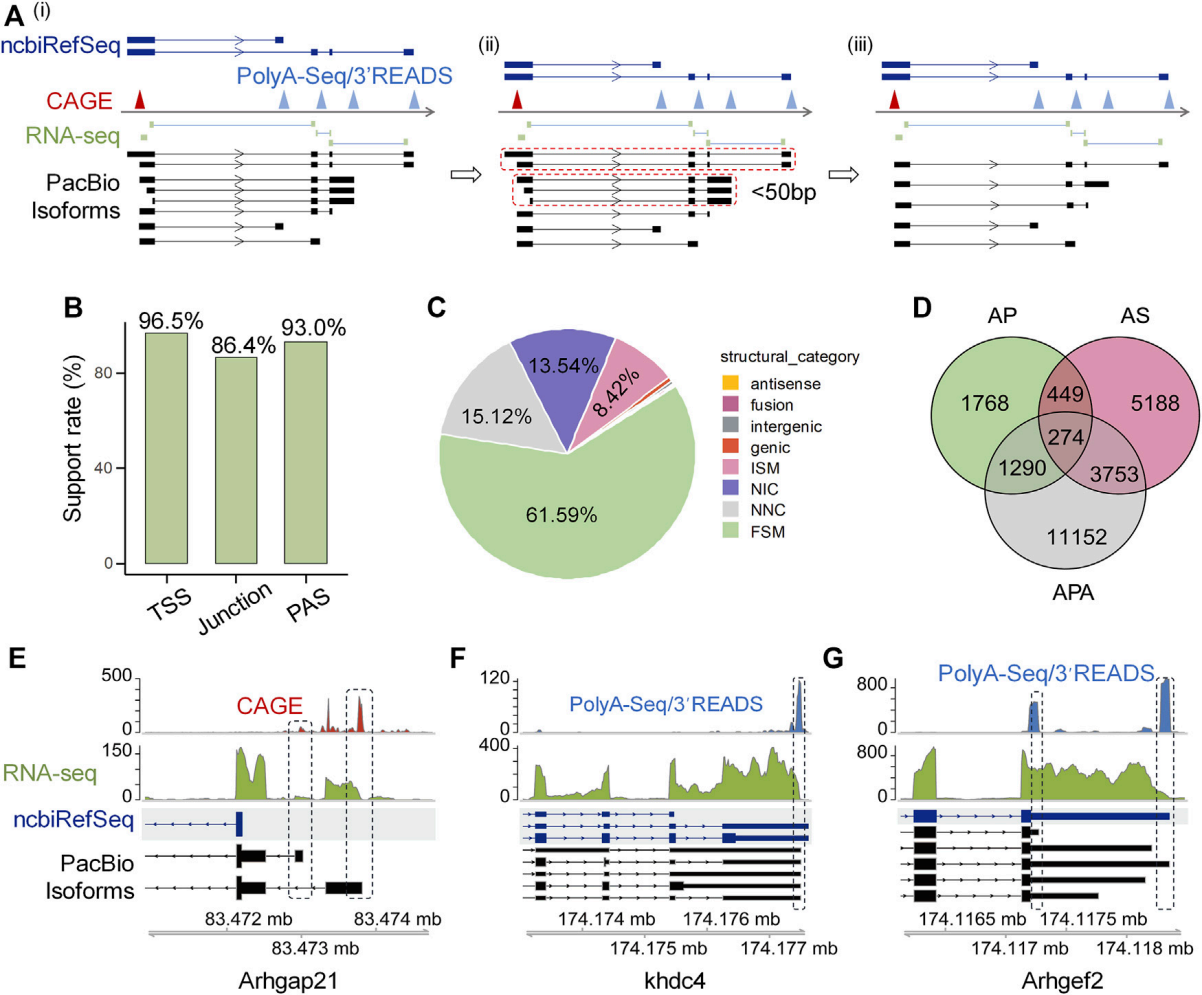
High confident full-length isoforms improve rat genome annotation. **(A)** The schematic diagram illustrating the cross-validation of full-length transcript end based on CAGE, PolyA-Seq and 3′READ. The transcriptome construction involves three steps. Step i: We generated validated pools of 5′-ends, splicing junction and 3′-ends from CAGE, short junction reads, PolyA-Seq and 3′READS, respectively, followed by comparing the aligned Iso-Seq isoforms with the validated 5′-end, junction, and 3′-end pool. Step ii: Mark those isoforms with the same splicing structure and PAS within 50 nt and only display 5′UTR difference (dotted closed circle). Step iii: We keep the one whose TSS is closest to the validated 5′-ends pool. **(B)** The support rate of PacBio isoforms for TSS, junction, and PAS independently. **(C)** Types and percentages of eight categories of PacBio defined transcripts. **(D)** Venn diagram shows the number of PacBio isoforms that differ from ncbiRefSeq in transcriptional start sites (TSS), alternative splicing (AS), and alternative polyadenylation (APA). **(E)** *Arhgap21* as an example to show novel isoforms with novel TSSs when compared to ncbiRefSeq and were supported by CAGE. **(F)** *Khdc4* as an example to show novel isoforms with intron retention when compared to ncbiRefSeq annotation and were supported by PacBio and RNA-seq. **(G)** *Arhgef2* as an example to show novel isoforms with proximal PAS when compared to ncbiRefSeq annotation and were supported by PacBio and PolyA-Seq/3′READS.

To validate the reliability of these isoforms identified by Iso-Seq, we used a series of public available datasets generated by independent methods including cap analysis gene expression (CAGE) data from FANTOM (Lizio et al., 2015), RNA-seq short junction reads, rat 3′READ raw data used in polyA_DB3 (Wang R. et al., 2018), rat PolyA-Seq data (Derti et al., 2012), and ncbiRefSeq known rat genome annotation (REF) to construct reliable 5′-ends, splicing junction, and 3′-ends pool, respectively (Figure 1Ai). If the isoforms have the same splicing pattern and PAS (within 50 nt) but different potential TSS, we only kept theose whose TSS are closest to the validated 5′-ends pool (Figure 1Aii, iii). Since Iso-Seq protocol uses the similar strategy as the Clontech SMARTer cDNA kit to generate full-length cDNA, which does not perform cap trap, and thus the obtained 5′ends can be possibly derived from degradation products or reverse transcriptase drop offs (Schaarschmidt et al., 2020). To filter out possible artificial isoforms, Iso-Seq defined transcription start site (TSS) and polyadenylation site (PAS) within 50 nt of validated 5′-ends and 3′-ends pool respectively, were called supported. As for splicing, if an Iso-Seq defined splicing event could be supported by at least two short junction reads in the pool, it was also called supported. We found considerable high support rates for TSS (96.5%), splicing junction (86.4%) and PAS (93.0%) (Figure 1B), suggesting the reliability of Iso-Seq detected isoforms.

After confirming the reliability of the detected isoforms, we next asked whether full-length Iso-Seq could improve the gene annotation of rat genome. We used UCSC ncbiRefSeq as a reference for comparison, because this annotation is the most complete for rat genome (Ji et al., 2020). We adopted the transcript structure classification strategy of SQANTI3 (Tardaguila et al., 2018), which divides transcripts into eight categories compared to reference, to classify the Iso-Seq identified transcripts (Supplementary Figure S2). Among all these transcripts annotated to known genes (*n* = 34,642), 61.59% (*n* = 21,337) were categorized as full-splice match (FSM, corresponds to a transcript with all the same splice junctions as a reference transcript) in catalog, 15.12% (n = 5,238) were categorized as novel not in catalog (NNC; transcripts use novel donors and/or acceptors), 13.54% (n = 4,692) were categorized as novel in catalog (NIC, transcripts contain new combinations of annotated splice junctions or novel splice junctions formed from annotated donors and acceptors), 8.42% (*n* = 2,916) were categorized as incomplete splice match (ISM, transcripts matching consecutive, but not all, splicing junctions of the REF), 0.59% (*n* = 204) were categorized as genic, 0.32% (*n* = 112) were categorized as intergenic transcripts, 0.23% (*n* = 80) were categorized as fusion, and 0.18% (*n* = 63) were categorized as antisense transcripts [structure categories defined by (Tardaguila et al., 2018)] (Figure 1C).

We proceeded to characterize transcripts in these eight structure categories above. First, we cross-validated the reliability of transcripts in each category. PacBio transcripts with 5′-ends within 50 nt to CAGE or REF TSSs peaks were considered as validated TSSs, and PacBio transcripts 3′-end within 50 nt to PASs (generated from PolyA-Seq, 3′READ or REF) were considered as cross-validated PASs. Junctions of PacBio transcripts supported with RNA-seq junction reads were used for percent calculation. In line with our speculation, FSM category has high validation rate regarding TSS (100%), splicing junction (99.87%) and PAS (92.98%) (Supplementary Figure S3). Noteworthy, two categories that contain novel sites (NNC) and (NIC) also showed consistently high validation rates (>95% for TSS, junction and PAS) (Supplementary Figure S3), suggesting the novel isoforms detected by Iso-Seq are reliable. For categories that have lower validation rates (such as junction in genic and antisense categories), experimental or technical errors could possibly account for them, as discussed previously (Tardaguila et al., 2018).

After demonstrating the authenticity of these novel isoforms identified by Iso-Seq, we further explored the potential underlying contributing factors. We found AP, AS and APA could all contribute to the generation of novel isoforms, and APA contributed most, followed by AS and AP (Figure 1D). Further, 92.83% of the APA-derived novel PASs are within 24 nt distance to validated PAS clusters (Supplementary Figure S4), further supporting the biggest contribution of APA to novel isoforms. There were several good examples showing novel isoforms annotated by our data. For instance, the gene *Arhgap21* has two novel TSSs not annotated in ncbiRefSeq but supported by CAGE data (Figure 1E). The gene *Khdc4* having intron retention (a type of AS), is also a contributor to novel isoforms (Figure 1F). The gene *Arhgef2* in the ncbiRefSeq annotation has only one PAS, however, we identified several APA isoforms of this gene based on our PacBio data, with both the shortest (novel proximal PAS) and the longest (annotated distal PAS) isoforms supported by strong PolyA-Seq/3′READs signals (Figure 1G). Together, these above results support the notion that a combination of Iso-Seq and RNA-seq could largely improve the rat genome annotation.

### Expression changes at both gene and isoform levels in Ang II-induced RAEC senescence

Previous analyses showed that Iso-Seq could identify full-length isoforms qualitatively but less quantitatively. In contrast, RNA-seq is more accurate in quantifying gene expression rather than distinguishing the different isoforms from the same gene (Huang et al., 2021). Having confirmed the improvement of rat genome annotation by the combination of Iso-Seq and RNA-seq, we further applied this strategy to explore the expression changes at both gene and isoform levels in Ang II-induced senescent RAECs. First, we used RNA-seq to perform differential gene expression analysis and identified a total of 2,671 differentially expressed genes (DEGs) between Ang II-induced RAECs and the Control ones, including 1,249 upregulated and 1,422 downregulated DEGs (Supplementary Figure S5A). The downregulated DEGs were most significantly enriched in Cell Cycle and DNA replication (Supplementary Figure S5B), consistent with the cell cycle arrest phenotypes in Ang II-induced senescent RAECs (Yang et al., 2020). The upregulated genes were highly enriched in ECM-receptor interaction, PI3K-Akt signaling pathway and TNF signaling pathway (Supplementary Figure S5C), which were known to promote senescence (Saito and Berk, 2002; Yin et al., 2003; Chen et al., 2012). Notably, a substantial number of DEGs were involved in Cell-Cycle pathway, including Cyclin- Dependent Kinase (CDK) gene family such as Cdk2, encoding a well-known cell cycle regulator, were decreased upon Ang II stimulation (Supplementary Figure S5D). DEGs of PI3K-Akt signaling pathway, such as those encoding the inflammatory cytokine IL-6 (IL-6), and vascular endothelial growth factors A and D (*Vegfa*, *Vegfd*) were increased upon Ang II stimulation (Supplementary Figure S5E). Overall, the DEG analysis with RNA-seq data indicates that the Ang II-induced cell senescence model is reliable and could be used for subsequent analysis.

**FIGURE 2:**
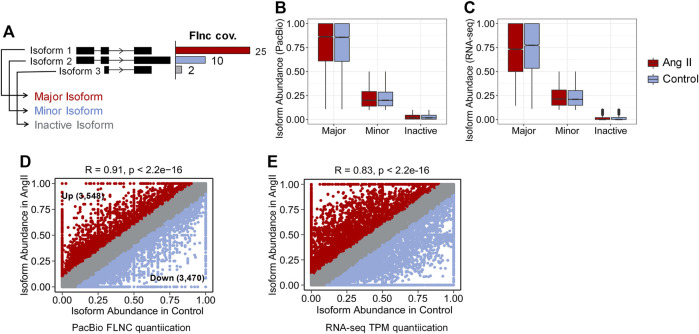
Isoform abundance changed between Control and Ang II. **(A)** Classification of isoform abundance according to the percentage of FLNC reads support. **(B)** Isoform abundance (PacBio quantification) distribution between Major, Minor and Inactive isoforms. **(C)** Isoform abundance (RNA-seq quantification) distribution between Major, Minor and Inactive isoforms. **(D)** Scatter plot of the isoform abundance between Ang II and control conditions (Iso-Seq quantification). (Up + Down)/(Total–Inactive) = (3,548 + 3,470)/(33,706–14,329) = 36.2%, Around 36.2% of the identified isoforms changed the proportion of usage before and after Ang II stimulation. **(E)** Scatter plot of the isoform abundance between Ang II and control conditions (RNA-seq quantification). Correlation of the isoform abundance (PacBio quantification) between Control and Ang II. The text in the figure indicates example genes.

Next, we used FLNC reads generated from Iso-Seq to perform isoform level analysis. FLNC reads were chosen because they can be considered as full-length isoforms (Wang et al., 2019). Although not as quantitative as RNA-seq, FLNC reads in Iso-Seq were still found to be semi-quantitative, especially for those full-length reads that derived from relatively highly expressed genes (Uapinyoying et al., 2020; Wyman et al., 2020). Therefore, we defined the isoform abundance as a percentage of isoform-supportive FLNC reads to the sum of all FLNC reads of a gene. The isoform with the largest percentage is called the major isoform, the isoform with abundance <0.1 is called inactive isoform, and the rest are called minor isoforms (Figure 2A). The abundance distribution of these three isoform types is in line with their definition (Figure 2B). The same trend was observed when quantification was performed with RNA-seq short reads (Figure 2C). We next compared the isoform abundance between Ang II and Control conditions. The isoform abundance between these two conditions was overall correlated (Figure 2D, Pearson’s correlation coefficient = 0.91; *p* < 2.2e-16). The abundance of most isoforms (78.62%, |Δ abundance| < 0.1) was not changed (Figure 2D). However, if we do not consider isoforms that are inactive before and after Ang II stimulation (*n* = 14,329) (Table 2), 36.2% of the isoforms undergo change (|Δ abundance| ≥ 0.1) in the proportion of usage before and after stimulation. Specifically, 3,548 isoforms showed upregulated expression and 3,470 isoforms showed downregulated expression upon Ang II treatment (Figure 2D). KEGG pathway enrichment analysis showed that genes with changed isoform usage were more likely to be enriched in senescence or aging-related pathways such as p53 signaling pathway, mTOR signaling pathway and MAPK signaling pathway when compared to those genes with unchanged isoform usage (Supplementary Figures S6A, B). We also used RNA-seq to examine the difference in isoform abundance and obtained similar results (Figure 2E; Table 3; Supplementary Figures S6C, D).

**TABLE 2:** State of isoform changing in Control and Ang II (Iso-Seq quantification).

Control.Class	Ang II.Class	*n*
Inactive	Inactive	14,329
Major	Major	8,651
Minor	Minor	4,559
Minor	Inactive	1,891
Inactive	Minor	1,813
Major	Minor	949
Minor	Major	915
Inactive	Major	340
Major	Inactive	259

**TABLE 3:** State of isoform changing in Control and Ang II (RNA-seq quantification).

Control.Class	Ang II.Class	*n*
Inactive	Inactive	22,469
Major	Major	6,737
Minor	Minor	1,132
Minor	Inactive	1,213
Inactive	Minor	1,194
Major	Minor	717
Minor	Major	751
Inactive	Major	1,359
Major	Inactive	706

We selected *Mxi1* and *Cdh2* as examples to show the isoform usage difference upon Ang II treatment, both genes were associated with aging/senescence diseases (Schreiber-Agus et al., 1998; Mayosi et al., 2017; Kruse et al., 2019; Zhuo et al., 2019). Iso-Seq detected three isoforms of *Mxi1*, and two of these isoforms showed differential expression between Ang II and Control, despite no differential gene expression (Log2FC = 0.16), highlighting the importance of examining isoform level changes (Supplementary Figure S7). *Cdh2* gene is widely studied in CVDs and other diseases (Mayosi et al., 2017; Kruse et al., 2019; Zhuo et al., 2019). *Cdh2* showed PAS preference after Ang II stimulation, the major isoforms of *Cdh2* in control tend to use the distal PAS while major isoforms in Ang II tend to use the proximal one (Supplementary Figure S8).

### Alternative promoter regulation in ang II-induced RAEC senescence

**FIGURE 3:**
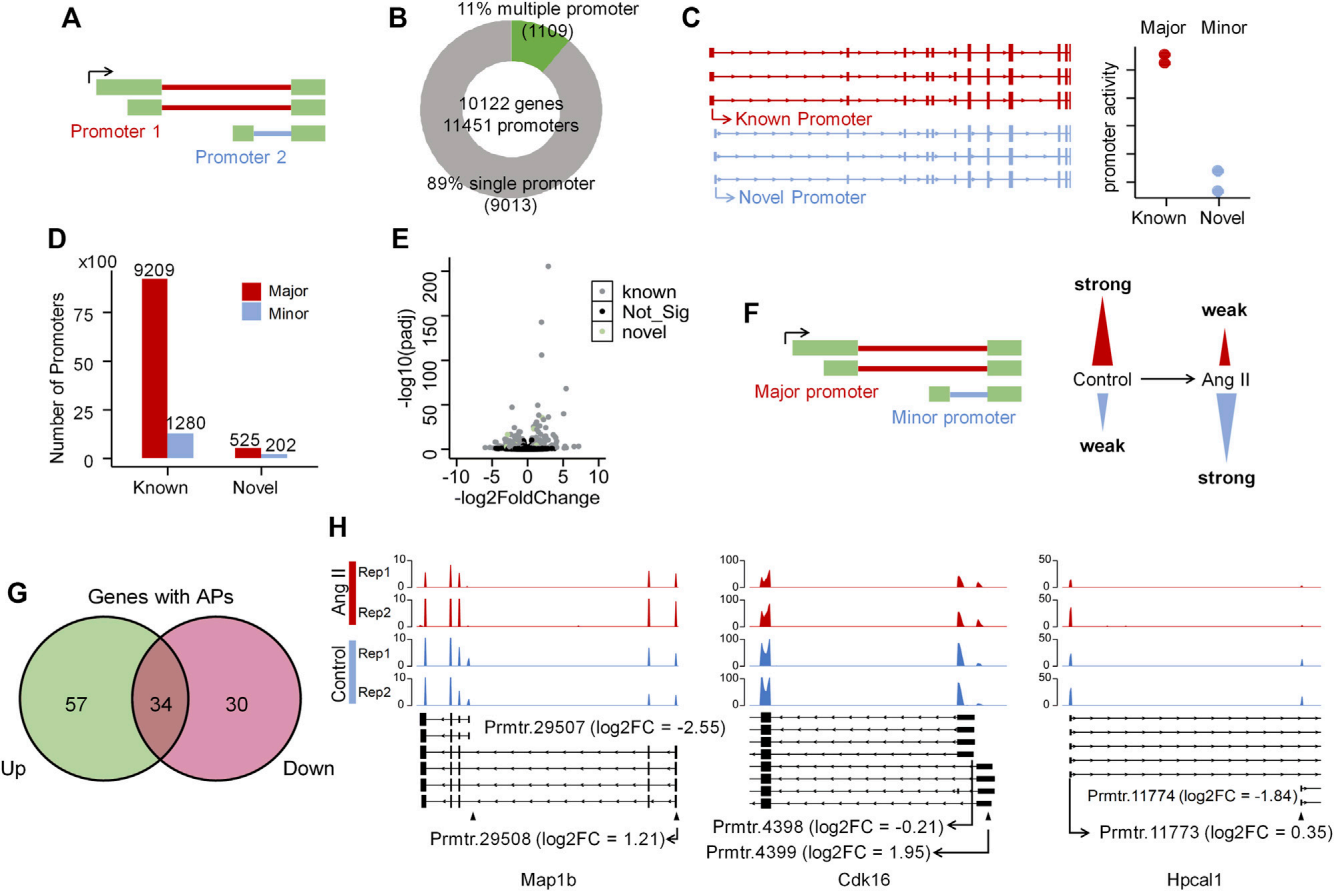
Alternative promoter activity analysis before and after Ang II treatment in RAECs. **(A)** Schematic representation of promoter definition in this study (Demircioğlu et al., 2019). **(B)** Number of single promoter genes and multiple promoter genes identified in the transcriptome that Iso-Seq generated. **(C)** Example of known and novel promoters in RAECs. Promoters are considered known if known ncbiRefSeq transcripts also use this promoter. Promoters with the highest average activity are considered Major promoters, and all other promoters of the same gene are assigned as Minor promoters (Huang et al., 2021). **(D)** The number of Major, Minor promoters in known and novel categories. Most promoters are known and the proportion of novel and minor promoters are small. **(E)** Differentially expressed promoter analysis in RAECs. Black color stands for not significantly differentially expressed promoters, gray color stands for significantly differentially expressed known promoters, and green color stands for significantly differentially expressed novel promoters. **(F)** Schematic representation of promoter activity switching. **(G)** Venn diagram shows the overlap of genes with upregulated (Up) and downregulated (Down) promoters. **(H)** Examples of genes that undergo dynamic changes in alternative promoter activity, alternative promoter was marked with ▲.

As alternative promoter (Demircioğlu et al., 2019; Huang et al., 2021), alternative splicing (Roy et al., 2013) and alternative polyadenylation (Chen et al., 2017) are three major contributors to isoform diversity in mammalian cells, we therefore respectively explored their contributions in Ang II-induced RAECs senescence. To explore the alternative promoter (AP) usage, we applied *proActive* (Demircioğlu et al., 2019) to carry out the promoter analysis, wherein the promoter usage was defined based on the first junction from transcriptome GTF file (here we used the GTF file generated from our Iso-Seq data), and the activity of the promoter was evaluated based on the number of junction reads supported by RNA-seq (Figure 3A). The promoter usage predicted by *proActive* is consistent with CAGE and H3K4me3 histone ChIP-seq data (Demircioğlu et al., 2019). We identified 1,109 genes with multiple promoters and 9,013 genes with single promoter (Figure 3B) using Iso-Seq data of RAECs. *proActive* classifies promoters into Major, Minor and Inactive according to their relative promoter activity. Promoters were also classified into known and novel promoters (Figure 3C, see Methods for details), and the known promoter accounted for the largest proportion, while the novel ones accounted for a small proportion (Figure 3D). We then applied DESeq2 to analyze differentially regulated promoters (DRPs) (Huang et al., 2021) between Control and Ang II-treated cells, and found 188 and 197 known promoters were upregulated and downregulated respectively in Ang II-treated RAECs. Notably, 18 and 10 novel promoters were upregulated and downregulated respectively upon Ang II stimulation (Figure 3E). Then we compared the genes with DRPs (DRPGs) with differentially expressed genes (DEGs) and found that 79.8% downregulated promoters overlapped with downregulated genes (Supplementary Figure S9A). These genes with downregulated promoters were enriched in functional categories such as cell division, nuclear division, negative regulation of cell cycle (Supplementary Figure S9B). 74.6% upregulated promoters overlapped with upregulated genes (Supplementary Figure S9C), and genes with upregulated promoters were enriched in Ang II stimulation-related terms such as cellular response to external stimulus, wound healing, regulation of vascular development, regulation of angiogenesis, and reactive oxygen species metabolic process (Supplementary Figure S9D). These results above indicated that the changed promoter expression may probably function in AngII-induced senescence in RAECs.

Next, we asked how many promoters are experiencing dynamic changes between Control and Ang II based on the promoter usage and relative promoter activity (Figure 3F). Relative promoter activity difference >0.1 and |FoldChange| > 1.5 were considered as alternative promoter (AP) between two conditions (Details in Methods section). A total of 239 APs derived from 121 genes were identified, including 34 genes with both upregulated and downregulated APs, which means switched promoter usage upon Ang II stimulation (Figure 3G). Three genes were selected as representatives for the promoter usage switch (Figure 3H). *Map1b* showed to have two promoters (Prmtr.29507 and Prmtr.29508) in Control, and after Ang II stimulation, Prmtr.29507 was downregulated (log2FC = −2.56) and Prmtr.29508 was upregulated (log2FC = 1.21) (Figure 3H left panel). In *Cdk16*, the inactive promoter (Prmtr.4399) in Control turned to be active after Ang II stimulation (log2FC = 1.95), though the major promoter (Prmtr.4398) kept not changed (log2FC = −0.21) (Figure 3H middle panel). As to *Hpcal1,* the activated promoter (Prmtr.11774) originally in Control turned to be inactive after Ang II treatment (log2FC = −1.84), but the major promoter (Prmtr.11773) did not show obvious change (log2FC = 0.35) (Figure 3H right panel). These observations suggest that APs serve as one contributor to the transcriptome diversity in Ang II-induced senescence in RAECs.

### Alternative splicing regulation in ang II-induced RAEC senescence

**FIGURE 4:**
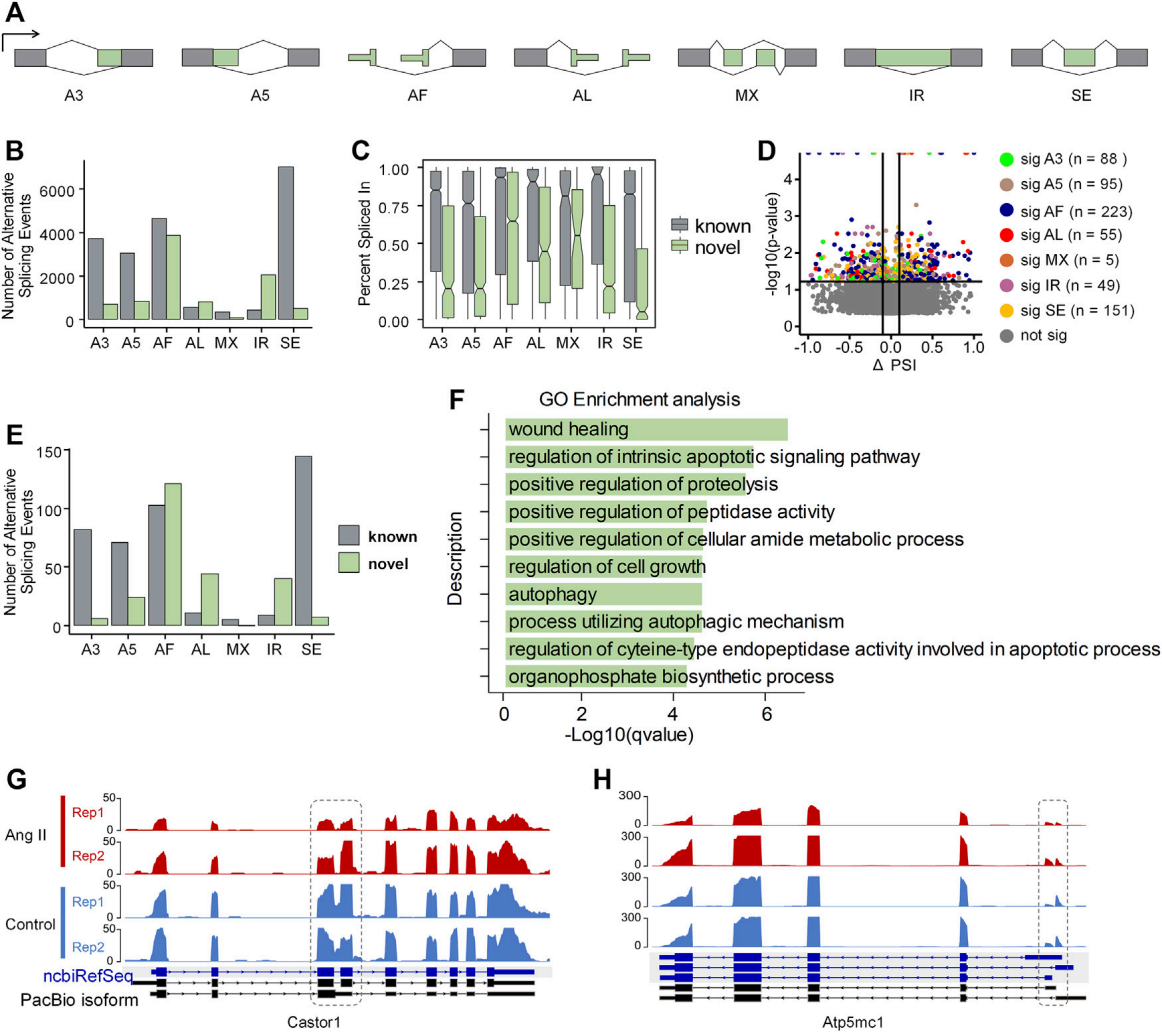
Overview of Alternative splicing (AS) events in Ang II-induced RAEC senescence. **(A)** Schematic diagram of the seven types of AS events defined by SUPPA2 when compared to ncbiRefSeq of rat genome. **(B)** The number of AS events detected by SUPPA2 based on Iso-Seq transcriptome. **(C)** Expression ratio of AS events in novel and known. **(D)** Differential AS events (*p*-value <0.05) between Ang II and control conditions. **(E)** The number of significantly spliced AS events between Ang II and control. **(F)** GO enrichment analysis of differentially spliced genes between Ang II and control cells. **(G)** RNA-seq track plot of gene *Castor1* before and after Ang II stimulation. Dashed circle indicates the differential intron retention (IR) event between two conditions. **(H)** RNA-seq track plot of gene *Atp5mc1* upon Ang II treatment. The dashed circle represents the alternative first exon (AF) event between two conditions.

Alternative splicing (AS) is a well-known contributor to transcriptome diversity, and participates in diverse biological processes (Wright et al., 2022). Therefore, we examined whether alternative splicing also showed dynamic changes upon Ang II stimulation in RAECs using SUPPA2 (Trincado et al., 2018). AS patterns were generated based on the GTF file generated from Iso-Seq and quantified by Salmon quantification results (See Methods for details). All the AS events were classified into seven types: alternative 3′-acceptor (A3), alternative 5′-donor (A5), alternative first exon (AF), alternative last exon (AL), mutually exclusive exon (MX), intron retention (IR) and exon skipping (SE) (Figure 4A). Among the expressed 10,122 genes based on PacBio Iso-Seq, 7,321 genes expressed more than one isoform. The most frequent AS type was SE (6,968), followed by AF (4,618), A3 (3,701), A5 (3,026), AL (593), IR (455) and MX (356) (Figure 4B). Interestingly, the most frequent novel AS type found was AF (3,877), and IR (2,047) was the second (Figure 4B), suggesting these two types of AS events are especially less annotated in the ncbiRefSeq. Next, we compared the percent spliced-in (PSI) values between known and novel in the seven AS types. We found that the expression of known AS events are generally higher than the novel ones. In novel AS events, the expression of AF, AL, MX was relatively higher than the expression of other events (Figure 4C). SUPPA2 was next used for identification of differential AS events between Ang II and Control, which was evaluated with ΔPSI, the difference of the mean PSI between Ang II and Control. There were 666 significant different AS events between Ang II and Control, among of which, AF counts the largest proportion (n = 223, 33.5%) (Figure 4D). Of note, AF was the most frequent novel differentially spliced AS type and SE was the most frequent known differentially spliced AS type (Figure 4E).

We functionally categorized the differential AS genes above and found that they were enriched in Gene Ontology (GO) terms such as wound healing, regulation of intrinsic apoptotic signaling pathway, and regulation of cell growth (Figure 4F). The importance of differential AS in Ang II-induced RAEC senescence is indicated by the following two example genes. *Castor1*, a regulator of mTORC1 (Li et al., 2021), which plays a key role in cell proliferation in response to nutrition and growth stimuli, showed decreased intron retention level (a novel AS event for Castor1) upon Ang II stimulation (Figure 4G). *Atp5mc1*, a gene associated with CVDs (Hartmann et al., 2022), also displayed a usage shift of alternative first exon upon Ang II treatment (Figure 4H). These results indicate that AS can contribute to Ang II-induced RAECs senescence through isoform changes in responsible genes.

### Alternative polyadenylation regulation in Ang II-induced RAEC senescence

**FIGURE 5:**
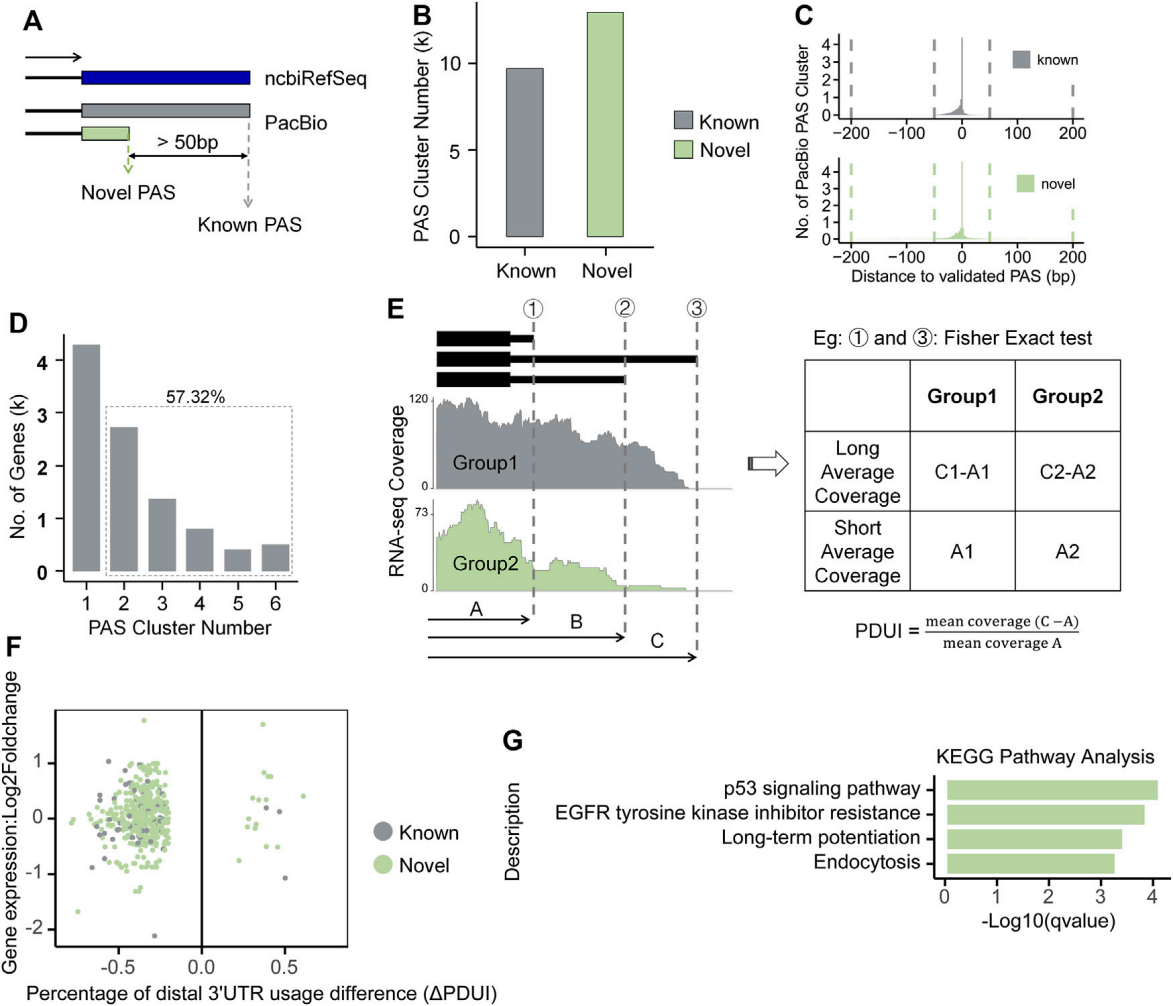
Global analysis of alternative polyadenylation combined with Iso-Seq and RNA-seq in RAECs. **(A)** The Schematic diagram of known and novel PASs. **(B)** The number of known and novel PASs identified by Iso-Seq. **(C)** Histogram of the genomic distance between Iso-Seq identified PASs and validated 3′ends pool in both known and novel classes. **(D)** Distribution of genes that have one or more PAS. **(E)** Differential usage of PAS was calculated based on given proximal PAS as prior knowledge. **(F)** Scatter plot between ΔPDUI and expression changes for genes with significantly longer or shorter 3′UTRs. Green and gray represent genes with novel and known PASs, respectively. **(G)** KEGG pathway enrichment analysis for genes tended to use proximal PAS after Ang II stimulation.

Alternative polyadenylation (APA) fine-tunes gene expression and largely enhances the complexity of mammalian transcriptome. To explore whether and to what extent APA contributes to the isoform changes in response to Ang II stimulation in RAECs, we performed APA analysis by integrating both Iso-Seq and RNA-seq data. We used Iso-Seq to identify Polyadenylation sites (PASs) and RNA-seq to quantify APA changes. PASs within 50 nt were clustered into one PAS, and PASs with distance >50 nt were considered as different PASs, as defined previously (Tang et al., 2022). If a PAS is within 50 nt of a known ncbiRefSeq PAS, it is considered as known, otherwise defined as novel (Figure 5A). We found that Iso-Seq detected a large number of PASs, including thousands of novel PASs (Figure 5B). To evaluate whether these novel PASs are authentic, we compared them with the experimentally verified 3′-ends pool generated by 3′READ, PolyA-Seq and polyA_DB3 of rat samples. We found that most PASs were within 50 nt to those in the validated 3′-ends pool for both known and novel PASs (Figure 5C). We analyzed the nucleotide composition of the 50 nt around the identified PASs and found that the 25 nt region upstream PAS is predominantly A-enriched, while the downstream is predominantly U-enriched (Supplementary Figure S10A), consistent with known literature reports (Chen et al., 1995). Moreover, we used MEME (Bailey et al., 2015) to discover motif and found the canonical polyadenylation signal (AAUAAA) also over-represented in the PASs (Supplementary Figure S10B) (Elkon et al., 2013). These above lines of evidence suggested that these PASs identified by Iso-Seq are of satisfied quality and reliable for further analysis. A total of 5,755 (57.32%) genes had at least two PASs based on Iso-Seq of RAECs (Figure 5D). We next analyzed the differential usage of PAS between Ang II and control RAECs using the modified algorithm of DaPars (Xia et al., 2014) for the differential PAS usage between Ang II and control RAECs. DaPars uses mean squared error (MSE) to predict the proximal PAS with mean RNA-seq coverage. Here Iso-Seq can provide reliable but not predicted PASs, we therefore used these PASs as prior knowledge and calculated their differential PAS usage between Ang II and Control cells by Fisher’s exact test (Figure 5E, see details in Method section). We found that 278 and 18 genes showed a preference usage of proximal and distal PASs, respectively, showing a global 3′UTR shortening upon Ang II stimulation (Figure 5F). Most of the length-changed 3′UTRs are derived from novel PAS (Figure 5F), suggesting Iso-Seq provides previously-unknown information for studying the APA regulation in Ang II-induced RAEC senescence. Further analysis showed that genes tending to use proximal PASs were enriched in pathways related to senescence such as p53 signaling pathway (Ciardiello and Tortora, 2008; Huang and Fu, 2015) (Figure 5G), implying that APA may fine-tunes certain key pathways to participate in regulation of Ang II-induced senescence.

As mentioned above, Iso-Seq and RNA-seq have their advantages and disadvantages. PacBio Iso-Seq data provides more reliable PASs even at relatively low coverage, and DaPars was originally designed to use Illunima’s RNA-seq data to predict proximal PASs usage and analyze global 3′UTR length changes between two conditions. Here we ran both the original DaPars program solely on RNA-seq data (Hereinafter referred to as RNA-seq) and the modified DaPars on both Iso-Seq and RNA-seq data (Hereinafter referred to as Iso-Seq), the results run with original DaPars used as comparison references. The DaPars result based on RNA-seq data also showed a global shortening trend of 3′UTR length after Ang II stimulation (Supplementary Figure S11A). DaPars using RNA-seq predicted a total of 260 differentially expressed proximal PASs, of which 119 were known ones (located within 50 nt to the reliable 3′-ends pool) (Supplementary Figure S11B). We found that most of the differential APA genes predicted by RNA-seq were also supported by Iso-Seq (Supplementary Figure S11C). In contrast, most of the differentially used APA events identified by Iso-Seq were not covered by RNA-seq, suggesting the necessity of integrating Iso-Seq and RNA-seq to achieve a more comprehensive APA results. *Pten* and *Pnkp* are two genes that were detected only in Iso-Seq but not in RNA-seq, their proximal PASs were supported by PolyA-Seq and 3′READ (Supplementary Figure S11D). *Pten* is a well-known tumor suppressor gene that functions in preventing cell survival and proliferation (Song et al., 2012). Our previous study discovered that SRSF3-mediated 3′UTR shortening of *Pten* could lead to senescence-associated phenotypes in both HUVEC and 293T human cells (Shen et al., 2019). Interestingly, this phenomenon is also evident in our Iso-Seq data in Ang II treated RAECs comparing to control ones (Supplementary Figure S12). *Pnkp* is a gene involved in DNA repair and in response to oxidative damage, it tends to use proximal PAS and therefore shortened 3′UTR after Ang II stimulation in RAECs (Supplementary Figure S11D). *Mpc1* was only detected to display 3′UTR shortening only in RNA-seq but not in Iso-Seq. Both the proximal and distal PASs of *Mpc1* were detected by PolyA-Seq and 3′READ, but Iso-Seq could only detect the distal one, possibly due to its limited sequencing coverage (Supplementary Figure S11D). These results demonstrate the power of integrating Iso-Seq and RNA-seq in comprehensive understanding of APA regulation in Ang II-induced RAEC senescence.

## Discussion

The rat genome annotation is far from complement compared to human and mouse (Wang et al., 2019). While mouse could serve as an important model to investigate the function of disease-related genes, rat has an irreplaceable role as a model organism to study cardiovascular diseases (CVDs) (Doggrell and Brown, 1998; Bader, 2010). Therefore, a more comprehensive annotation of gene structures in the rat genome would greatly facilitate study in related fields. RNA-seq has been commonly used for differential gene expression analysis and transcript structure construction. However, the major limitation of short reads-based RNA-seq strategy makes it difficult to construct full-length transcripts by simply assembling those short RNA-seq reads (Abdel-Ghany et al., 2016). Compared with short-read sequencing, long-read sequencing such as PacBio single-molecule real-time (SMRT) sequencing (Iso-Seq), has the advantage of relatively long read length and therefore can effectively overcome the above limitation. Iso-Seq can capture full-length isoforms without the need of further assembly, which makes it an effective method for analyzing gene structure at isoform level. Nevertheless, Iso-Seq has relatively low sequencing depth and is not as quantitative as RNA-seq, therefore, a combination of both Iso-Seq and RNA-seq would improve the rat genome annotation and quantify the critical changes at isoform level. In additon to integratively analyzing Iso-Seq and RNA-seq data, we also evaluated the reliability of the PacBio isoforms with the help of CAGE, short junction reads, PolyA-Seq, 3′READS data, and found that most of the identified transcripts were reliable (Figure 1B). By integrating all these types of data, we identified a total of 13,305 novel isoform structures, which greatly improve the rat genome annotation. To the best of our knowledge, this is the first study to combine both PacBio Iso-Seq and Illumina RNA-seq to reveal both transcrtional and post-transcriptional regulation in the Ang II-induced RAEC senesence model.

Alternative promoter (AP), alternative splicing (AS) and alternative polyadenylation (APA) are three major contributors to isoform diversity at either transcription or post-transcription level. Different isoforms of the same gene can contribute differently to physiological and developmental processes (Graveley, 2001; Baralle and Giudice, 2017). We demonstrated that integrating Iso-Seq and RNA-seq could reveal the contribution of these three types to transcriptome diversity.

Around 36.2% of the identified isoforms showed changed usage before and after Ang II stimulation (Figure 2D), and some of them were associated with key genes related to Ang II stimulation-associated phenotypes. Genes with isoform usage shift were enriched in cellular senescence related pathways such as MAPK signaling pathway, mTOR signaling pathway. For example, *Mxi1* is an antagonist of *Myc*, a master regulator of cell proliferation and tumorigenesis (Engstrom et al., 2004). The previous study revealed that isoform Mxi1-0, but not Mxi1-1, was highly expressed in pulmonary arterial smooth muscle cells of hypoxic pulmonary hypertension patients, and only Mxi1-0 was induced by hypoxia and plays an essential role in cell proliferation (Dong et al., 2022). Our Study found that *Mxi1* did not show significantly differential expression upon Ang II stimulation (log2FC = 0.16, FDR = 0.54), but two of its three isoforms were specific in Control and Ang II (Supplementary Figure S7), suggesting that Ang II stimulation induced the switched isoform usage but not the total gene expression change for certain genes. Such switched isoform usage would not be easily discovered solely based on regular RNA-seq data. Further examination indicated that exon skipping (one of the dominant forms of alternative splicing) contribute to the isoform usage shift in *Mxi1* upon Ang II treatment (Supplementary Figure S7). *Cdh2*, a gene widely studied in CVDs and other diseases (Mayosi et al., 2017; Kruse et al., 2019; Zhuo et al., 2019), demonstrates APA in control and Ang II (Supplementary Figure S13). Loss- or gain-of-function analysis showed that CDH2 can significantly promote angiogenesis and sensitivity to the antagonist exherin both *in vitro* and *in vivo* (Zhuo et al., 2019). Mutations in *CDH2* has been widely studied in arrhythmogenic right ventricular cardiomyopathy (ARVC) (Mayosi et al., 2017), but the effects of APA of *Cdh2* have not been reported and worth further study.

We used *proActive* to study the change in alternative promoter usage in the system of Ang II-induced RAECs cellular senescence, the result showed that most genes only use one promoter. Genes with differentially regulated promoters (DRPGs) is much fewer than the differentially expressed genes (DEGs), and ∼85% DRPGs overlap with DEGs (n = 308) (Supplementary Figure S9). A large number of DEGs were not covered by DRPGs. Both DEGs and DRPGs were analyzed using DESeq2, and we note that one difference in this count is that the DRPG analysis was done using the first junction reads of the promoter, which is an order of magnitude smaller than the number of reads used for the DEG analysis. Although there is a large variation regarding the FoldChange of the junction reads. But DESeq2 does not consider the *p*-value to be significant. Interestingly, although the number of DRPGs is relatively small, they can perform a critical function. For example, genes with down-DRPGs were enriched in cell division and those with up-DRPGs enriched in cellular response to external stimulus, suggesting that DRPGs play a role in Ang II stimulated cells. Besides, we also investigated the alternative promoter (AP), and identified 239 APs derived from 121 genes, among of which are several cellular senescence-associated genes, such as *Map1b*, *Cdk16*, and *Hpcal1*. Of note, our study is the first to report the AP regulation in Ang II-stimulated RAEC senescence model. Previous results demonstrated that the phosphorylation of MAPs (including MAP2 and MAP1B) and MAP1B protein level were modulated by AT2R (Ang II type 2 receptor) upon Ang II stimulation (Gallo-Payet et al., 2011). In the present study, we first found that Ang II stimulation of RAECs induces promoter usage shift in *Map1b* (Figure 3H left panel). Similarly, we also found the activation of the minor promoter of *Cdk16* after Ang II stimulation for the first time (Figure 3H middle panel), implying the importance of AP regulation in cellular senescence.

Recent large-scale studies have illustrated the importance of AS in cellular senescence (Blanco and Bernabéu, 2012; Bhadra et al., 2020). The present study also discovered AS events in Ang II-induced RAEC models. The most frequent AS events are AF and SE, in addition, Iso-Seq identified a lot of novel alternative first exon (AF) and intron retention (IR) events (Figure 4B). The extensive AS indicates the complexity of cellular response to Ang II stimulation. Some of the differential AS are of genes with crucial roles in senescence or response to Ang II stimulation. For example, we identified a novel and downregulated IR events in *Castor1* after Ang II stimulation (Figure 4G). Studies have reported the importance of *Castor1* in the mTOR complex I (mTORC1) signaling pathway (Gai et al., 2016) and RNF167 activates mTORC1 and promotes tumorigenesis by targeting CASTOR1 for ubiquitination and degradation (Li et al., 2021), but little is known about the functional role of IR in *Castor1*. Another example is *Atp5mc1*, the gene encoding a subunit of mitochondrial ATP synthase and associated with CVDs (Hartmann et al., 2022), showed differential AF events in Ang II treated RAECs (Figure 4H).

There are several methods utilizing RNA-seq to detect potential PAS and differential APA such as DaPars (Xia et al., 2014), APAtrap (Ye et al., 2018), QAPA (Ha et al., 2018), TAPAS (Arefeen et al., 2018), and ChangePoint (Wang et al., 2014), while TAPIS (Abdel-Ghany et al., 2016) is an APA analysis pipeline that only uses Iso-Seq. However, none of the above-mentioned methods combines the advantage of Iso-Seq and RNA-seq. Iso-Seq can supply with numerous relatively reliable PAS and RNA-seq can be used for their quantification. Notably, novel PASs identified by Iso-Seq in our study even overnumbered the known PASs. The modified DaPars predicted Iso-Seq PAS as well as those predicted by the original DaPars showed a global shortening of 3′UTR in Ang II-stimulated RAECs (Figure 5F; Supplementary Figure S11A). *Pten*, a famous tumor suppressor gene that controls a wide range of cellular functions such as survival, proliferation, energy metabolism, and cellular architecture (Song et al., 2012), had been reported to be regulated by the splicing factor SRSF3, whose downregulation could cause the use of proximal PAS of *Pten* and finally lead to cellular senescence (Shen et al., 2019). We found similar proximal PAS usage preference of *Pten* in Ang II-induced RAEC senescence by analyzing Iso-Seq data (Supplementary Figure S12). However, such APA regulation was missed by the original DaPars that only using RNA-seq data. There were many other APA genes identified by original DaPars and modified DaPars such as gene *Dazap1*, *Dcn*, and *Cd2ap* (Supplementary Figure S11D), which were reported to be associated with cellular senescence (Saurus et al., 2017; Wright et al., 2017; Woldhuis et al., 2020). Altogether, the results in the present study implied that APA-mediated 3′UTR shortening may also play a role in Ang II-induced senescence in RAECs.

## Conclusions

Overall, the present study systematically surveyed the transcriptome changes at isoform level in Ang II-induced senescent RAECs by integrative analysis of corresponding Iso-Seq and RNA-seq data. Based on Iso-Seq-identified full-length transcripts, we discovered molecular changes at different levels including AP, AS and APA in Ang II-induced RAEC senescence. Genes with these alternative usages were found having senescence-related functions or biologically explicable for Ang II induced phenotypes, providing new clues to understand the molecular and regulatory mechanisms underlying cell responses to Ang II. Moreover, due to the long-length nature of Iso-Seq, the results of the present study also greatly improve the annotation of the rat genome, providing a valuable source for study with rats.

## Data Availability

The original contributions presented in the study are publicly available. This data can be found here: https://www.ncbi.nlm.nih.gov/search/all/?term=PRJNA889675.

## Supplementary material

Supplementary Figure S1 PacBio data analysis pipeline.

Supplementary Figure S2 The schematic diagram of eight transcript categories classified by SQANTI3.

Supplementary Figure S3 Cross-validation of eight categories of PacBio transcripts with public data. **(A)** TSS supporting rate of eight categories of PacBio transcripts. **(B)** RNA-seq junction supporting proportion of eight categories of PacBio transcripts. **(C)** PAS supporting rate of eight categories of PacBio transcripts.

Supplementary Figure S4 Distribution of the distance of novel PASs to validated PAS cluster.

Supplementary Figure S5 Differentially expressed genes in RAECs before and after Ang II stimulation. **(A)** The volcano plot showing the number of DEGs with a fold-change threshold of 1.5 and the adjusted *p*-value threshold of 0.05. extremely remarkable genes and some important genes were marked with text. **(B)** Top pathways enriched with down-regulated genes in Ang II-treated RAECs. **(C)** Top pathways enriched with up-regulated genes in Ang II-treated RAECs. **(D)** Heatmap of differentially expressed genes in Cell-Cycle pathway. **(E)** Heatmap of differentially expressed genes in PI3K-Akt pathway.

Supplementary Figure S6 KEGG analysis of genes whose transcript usage changes between Control and Ang II. **(A)** KEGG analysis was performed on transcripts quantified by PacBio with unchanged usage status. **(B)** KEGG analysis of PacBio quantified transcripts with changed usage status. **(C)** KEGG analysis of RNA-seq quantified transcripts with unchanged isoform usage status. **(D)** KEGG analysis of RNA-seq quantified transcripts with changed usage status.

Supplementary Figure S7 PacBio FLNC reads track of *Mxi1* in Control and Ang II-treated RAECs.

Supplementary Figure S8 PacBio FLNC reads track of *Cdh2* in Control and Ang II-treated RAECs.

Supplementary Figure S9 Differentially regulated promoters in RAECs before and after Ang II stimulation. **(A)** Venn diagram showing the relationship between down-regulated genes and differential promoters. **(B)** GO enrichment analysis result for down-regulated promoters. **(C)** Venn diagram showing the relationship between up-regulated genes and differential used promoters. **(D)** GO enrichment analysis result for up-regulated promoters.

Supplementary Figure S10 Sequence features of PacBio identified PASs. **(A)** Distribution of nucleotide component 50 nt around PAS, 0 denotes the position of PAS. **(B)** Motif analysis on 40 nt sequence upstream PAS by MEME.

Supplementary Figure S11 Length changes in 3′ UTR based on RNA-seq analysis. **(A)** Scatter plot between ΔPDUI and expression changes for genes with significantly longer or shorter 3′ UTR. **(B)** Histogram showing the distribution of genomic distance between RNA-seq predicted significantly proximal PASs and those in 3′-ends pool. There are 260 PASs predicted to be significantly longer or shorter and 119 of these are within 50 nt distance to those in the 3′-ends pool. **(C)** Venn diagram showing the relationship between Dapars analysis result based on PacBio data and RNA-seq data. **(D)** Example of genes tending to use proximal PAS after Ang II stimulation.

Supplementary Figure S12 PacBio FLNC track of *Pten*. From this track we can see that *Pten* tends to use the proximal PAS in Ang II-treated RAECs.

Supplementary Figure S13 RNA-seq track for gene *Cdh2*.
