# Supplementary figures and images for: Integrative analysis of Iso-Seq and RNA-seq reveals dynamic changes of alternative promoter, alternative splicing and alternative polyadenylation during Angiotensin II-induced senescence in rat primary aortic endothelial cells
Source: Front Genet. 2023 Jan 19;14:1064624. doi: 10.3389/fgene.2023.1064624 (PMC9892061; doi:10.3389/fgene.2023.1064624)

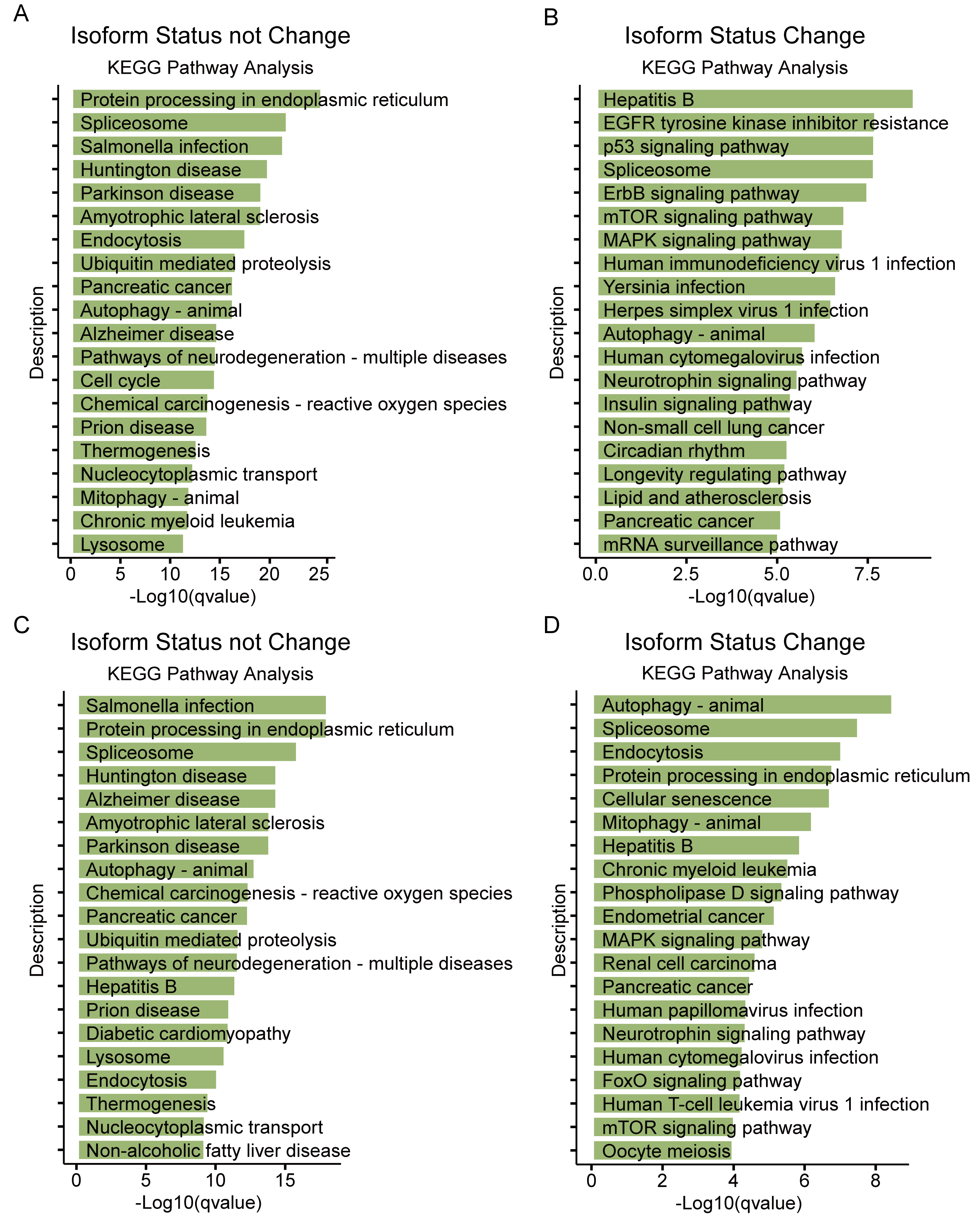

Supplement: Supplementary file 1 [file Image6.TIF]
